# Partitioning All-Cause Pre-Analytical Laboratory Rejections: A 25-Month Retrospective Audit of Specimen Quality Failures with Six Sigma, IFCC, and Pareto Analysis

**DOI:** 10.3390/jcm15197663

**Published:** 2026-10-03

**Authors:** Shoukat Ali Arain, Abdullah Naji Alhawil, Shahid Akhtar Akhund, Mahnoor Ali, Salman Aldosari, Abdulwahab Z. Binjomah

**Affiliations:** 1 Department of Pathology, College of Medicine, Alfaisal University, Riyadh 11533, Saudi Arabia; shali@alfaisal.edu; 2 College of Medicine, Alfaisal University, Riyadh 11533, Saudi Arabia; aalhawil@alfaisal.edu (A.N.A.); mali03@alfaisal.edu (M.A.); salmaldosari@alfaisal.edu (S.A.); abinjomah@alfaisal.edu (A.Z.B.); 3 Department of Medical Education and Anatomy and Genetics, College of Medicine, Alfaisal University, Riyadh 11533, Saudi Arabia; 4 Bacterial Diseases & Special Pathogens, Public Health Authority, Riyadh 13352, Saudi Arabia

**Keywords:** IFCC quality indicators, Pareto analysis, pre-analytical errors, pre-analytical quality failures, rejection events, Six Sigma

## Abstract

**Objectives:** Aggregate pre-analytical rejection rates conflate specimen-quality failures and administrative events, which require distinct mitigation protocols. We disaggregated rejection events to isolate specimen-quality failures and benchmarked them against global frameworks. **Methods:** We conducted a 25-month retrospective audit (July 2023 to August 2025) of test orders received by the Al Rass General Hospital laboratory. Using an AI-assisted classification protocol, we isolated pre-analytical Specimen Quality Failures from operational and administrative events. We mapped Specimen Quality Failure causes into IFCC WG-LEPS Quality Indicators (QIs) and evaluated via Six Sigma metrics, IFCC benchmarks, and Pareto analysis—stratified by laboratory section, sample type, priority, and work shift. **Results:** Of 905,018 test orders received, 35,476 were rejected (overall rejection rate 3.92%). However, the specimen-quality failures were 2.21% (σ = 3.51), with 15,495 entries excluded as operational events. Clotted samples (26.2%), hemolysis (23.0%), wrong sample type (20.2%), sample not received (7.8%), and insufficient volume (7.1%) together accounted for 84.3% of specimen quality failures (the Vital Few). Most individual categories scored Good (≥4.0) on Sigma, despite a pooled Sigma of 3.51 (Acceptable). Benchmarking against IFCC WG-LEPS targets showed that 6 of 13 quality indicators, including clotted sample, wrong sample type, and insufficient volume, fell Below Minimum, demonstrating divergent absolute internal process capability metrics and population-relative external benchmark performance signals. STAT samples had a lower specimen quality failure rate than Routine samples (1.25% vs. 2.51%). Although the morning shift accounted for 80.98% of total rejections, only 53.4% represented true specimen quality failures. **Conclusions:** Headline laboratory rejection rates are inflated by operational and administrative events, necessitating partitioned reporting models. While individual Westgard Sigma scores demonstrate satisfactory absolute process capability across most specimen quality failure categories, cross-referencing against population-relative IFCC targets contextualizes these results and identifies areas performing in the lower international quartiles. Quality improvement strategies require structural changes and workforce training.

## 1. Introduction

Clinical laboratories generate fundamental data driving the majority of medical decisions, making testing process integrity inseparable from patient safety [1]. This journey is best conceptualized as a continuous “brain-to-brain” loop, spanning the Total Testing Process (TTP) from a clinician’s initial diagnostic question to the final clinical action [2].

While automated analyzers and internal quality control mechanisms have minimized analytical error gaps, the pre-analytical phase remains heavily dependent on manual handling. Consequently, this phase accounts for approximately 46% to 68% of all laboratory errors. The most visible consequence of these errors is sample rejection due to hemolysis, clotting, insufficient volume, incorrect containers, or labeling mistakes. This leads to delays in diagnoses and clinical management, resulting in high institutional costs [3,4,5]. Since these events are logged by the Laboratory Information System (LIS) electronically, the rejection rate is recognized as a standard model Quality Indicator (QI) by the IFCC Working Group on Laboratory Errors and Patient Safety (WG-LEPS) [6].

Published pre-analytical rejection rates vary widely worldwide. A comprehensive meta-analysis reported variation across studies, with a pooled average of 1.99% [7]. Mirroring this global heterogeneity, regional audits across Saudi Arabia reveal similarly stark variations, ranging from 0.065% [8] to 12.1% [9]. A critical analysis of the literature reveals that conventional, all-cause rejection reporting suffers from substantial confounding by grouping structurally distinct failure modes. To establish an unconfounded baseline, these events must be segregated into two independent domains based on three systemic factors:

Mechanistic Conflation: True specimen defects, such as hemolysis and clotting, dominate pre-analytical errors and represent the primary actionable targets for frontline training. Unsegregated reporting forces these technical errors to compete with administrative faults [9,10], preventing an accurate assessment of frontline staff behavior under the Westgard Six Sigma or IFCC WG-LEPS frameworks.

Benchmarking Distortion: Wide variations in published rejection rates are heavily driven by how much an institution’s aggregate data is skewed by hidden operational faults rather than true collection competency [9,11]. Without formal segregation, cross-institutional quality benchmarking remains fundamentally unaligned.

Automation Vulnerabilities: Because automated laboratory systems cannot inherently distinguish frontline specimen collection compromises from systemic logistical faults, a setting-specific audit that decouples these two categories is an absolute prerequisite. This step filters background institutional noise, enabling precise quality target-setting, accurate application of Pareto frameworks to identify the Vital Few root causes, and the design of targeted operational interventions.

To establish a precise and unconfounded quality baseline, this investigation systematically segregated all rejection events into two operationally independent domains using a single, specimen-dependent separating criterion: Pre-Analytical Specimen Quality Failures (frontline collection and handling compromises) and Operational and Administrative Non-Performance Events (logistical and infrastructure faults). The present study reports exclusively on Pre-Analytical Specimen Quality Failures, which represents the true specimen-dependent fraction.

Consequently, this study audited all diagnostic test orders received by the clinical laboratory to characterize this specific failure domain. Our specific objectives were threefold: first, to quantify the overall prevalence of pre-analytical rejections due to specimen quality failures and identify the predominant rejection causes within this facility; second, to benchmark institutional process capability against international quality specifications using both the Westgard Six Sigma and IFCC WG-LEPS frameworks; and third, to evaluate variations in rejection rates and error profiles stratified across specific laboratory sections, work shifts, and clinical priority designations (Routine versus STAT).

By applying these complementary analytical frameworks, this investigation provides a granular, unconfounded baseline of frontline pre-analytical performance. Ultimately, these findings seek to identify precisely where targeted quality improvement interventions, policy refinements, and educational initiatives will yield the greatest operational efficiency and clinical benefit.

## 2. Materials and Methods

### 2.1. Study Design and Setting

This study was designed as a retrospective observational audit covering 25 months from July 2023 to August 2025. The research was conducted within the clinical laboratory at Al Rass General Hospital (Qassim Health Cluster, Kingdom of Saudi Arabia), a multi-specialty automated diagnostic facility encompassing 12 sections: Chemistry, Hematology, Hormones & Virology, Hemostasis, Parasitology, Point-of-Care Testing (POCT), Microbiology, Special Proteins/Tumor Markers, Serology, Blood Bank, Pathology, and Drugs/Toxicology.

### 2.2. Sample Selection and Unit of Analysis

All diagnostic test requests logged within the LIS during the 25-month study window were evaluated for eligibility, regardless of final transactional status (completed or rejected). Records with incomplete or unresolvable identifying metadata that could not be reliably mapped to a specific laboratory section or sample matrix were excluded. The sample matrix encompassed blood (serum, plasma, and whole blood), urine, stool, swabs, body fluids, and tissue/organ specimens for histopathological examination. To ensure analytical integrity, structural inclusion and exclusion criteria were applied.

In clinical laboratory operations, a single physical tube or collection container can generate multiple distinct test orders, each logged as a separate line entry. Because completed records lacked unique physical container tracking information to reliably collapse multi-test orders back to individual tubes, the primary unit of analysis throughout this study is the test order record rather than unique physical specimen.

### 2.3. Data Extraction and Cohort Engineering

Data were extracted electronically from the LIS. For each record, the dataset captured the patient identifier, receiving laboratory section, sample type, requested clinical test, order priority (Routine vs. STAT), final collection status, and the reason for rejection.

A deduplication step was applied specifically to rejected entries: multi-row duplication of a single rejection event, arising when several tests were ordered on the same rejected specimen and each generated its own log entry, was identified and collapsed using an AI-assisted, deterministic data-cleaning protocol (Claude Sonnet 5, Anthropic, San Francisco, CA, USA), matching on patient identifier, documented rejection reason, and rejection timestamp. This step removed 282 duplicate entries. The resulting dataset retained test/exam-order granularity (one row per test ordered) rather than a fully collapsed unique-specimen count. The final analytic cohort comprised 905,018 records, consisting of 869,542 completed test orders and 35,476 rejected test-order entries.

### 2.4. Data Partitioning, AI Mapping, and Governance Tiers

To process the raw, free-text LIS rejection logs, a deterministic, auditable mapping dictionary was constructed using Claude Sonnet 5 to review the 98 distinct raw rejection-reason strings in the dataset (Supplementary File S1). Where a raw string did not correspond to a representative example explicitly provided in the classification framework (38 of the 98 distinct strings, accounting for 455 of the 35,476 total rejection entries, 1.3%), these cases were flagged for manual review. The Principal Investigator reviewed and resolved each flagged classification in consultation with the AI system, strictly applying the framework’s stated inclusion criteria. The remaining 35,021 rejection entries (98.7%) matched an established framework example verbatim and required no manual disambiguation.

This finalized mapping dictionary was then applied across all rejection entries to execute a two-stage categorical assignment:

Stage 1—Selection of Specimen Quality Failures: To isolate specimen quality failures from background administrative noise, rejection records were evaluated using a single, binary selection criterion: Would a perfectly collected and handled specimen still have been rejected for the same documented reason? Only entries where the answer was “no”, indicating that the biological specimen itself was compromised, were included as Specimen Quality Failures. Entries where the answer was “yes”, indicating rejection due to systemic, administrative, or resource factors regardless of specimen quality, were classified as Operational and Administrative Events and excluded from primary analysis.

Stage 2—QI sub-classification: Specimen quality failure causes were mapped into 15 Quality Indicator (QI) categories (QI-1 through QI-15), consistent with the IFCC WG-LEPS taxonomy, where applicable [12,13].

### 2.5. Validation of the Classification and Audit Procedure

Sample Selection and Independent Manual Verification: To verify classification accuracy, an audit subsample comprising approximately 10% of entries within each QI category was selected. Sampling was stratified by QI code to ensure proportional representation across all 15 categories and prevent the under-sampling of rarer error types. In addition, all 98 distinct raw rejection-reason strings present in the dataset, including all 38 variants requiring manual disambiguation, were systematically audited. Two independent authors (ANA and MA), both familiar with the classification framework and coding rules, audited the coded dataset while blinded to each other’s assessments. Agreement was defined as exact concordance between the reviewer’s independent clinical determination and the assigned code. Across all audited entries, both reviewers exhibited 100% raw concordance with the automated mapping (zero discrepancies identified), rendering formal third-party adjudication or chance-corrected inter-rater statistics inapplicable.

Statistical Rationale for Sampling Fraction: A formal a priori sample size power calculation was not performed because classification was executed via a deterministic mapping dictionary rather than unconstrained, stochastic row-by-row natural language inference. Each unique raw text string was assigned to an indicator category once and subsequently propagated identically across all occurrences. Consequently, classification error in this architecture is a per-string rather than a per-row phenomenon; verifying 100% of the 98 distinct raw strings and their underlying rules verify the mapping logic across the entire cohort.

Pipeline Reproducibility: Reproducibility was confirmed by re-executing the end-to-end data-cleaning and classification pipeline across three independent, isolated computational sessions. The resulting outputs demonstrated complete mapping stability, with only minor, non-material variances (<0.01%) observed, attributable exclusively to the deduplication matching threshold rather than the classification logic itself.

### 2.6. Quality Benchmarking Frameworks

Specimen quality failures were benchmarked using two complementary analytical frameworks, applied across laboratory sections, sample types, and clinical priority tiers:

Westgard Six Sigma Metrics: Sigma values were calculated using the standard long-term (1.5σ-shifted) conversion widely used in laboratory Six Sigma applications [14]: Sigma = Φ^−1^(1 − defect rate) + 1.5, where Φ^−1^ is the inverse standard normal cumulative distribution function and the defect rate is expressed as observed defects per million opportunities (DPMO). This is the conventional scale where 6.0 represents world-class performance (≈3.4 DPMO). Process capability was classified as World Class (≥6.0), Excellent (5.0–5.9), Good (4.0–4.9), Acceptable (3.0–3.9), or Needs Improvement (<3.0) [14]. Conversion reference tables are provided in Supplementary File S2.

IFCC WG-LEPS Benchmarks: Where available, observed performance was categorized into Optimal, Desirable, or Below Minimum tiers using verified specifications published by the IFCC WG-LEPS [13]. Rather than static theoretical limits, these specifications are derived empirically from the distribution of results reported by international participating laboratories within a continuous, multi-cycle quality-indicator project, wherein the 25th, 50th, and 75th percentiles establish the thresholds for Optimal, Desirable, and Below Minimum performance, respectively. The 2017–2018 cycle served as our primary benchmark [13]; to evaluate the robustness of our results, we also performed a sensitivity analysis using the subsequent 2021 cycle [15].

The 2017–2018 dataset reports only percentage-based quality specifications, without Sigma values. The 2021 dataset reports a “short-term sigma value” (STSV), which is conventionally calculated without the 1.5σ shift applied in our own formula, so the two values may not be directly comparable. Thus, we conducted both the primary and sensitivity benchmark comparisons on the percentage defect-rate scale.

QI specifications within the IFCC WG-LEPS model are defined relative to specific denominators. In seven of thirteen benchmarkable QIs, we used denominators identical to the IFCC specification (total samples received). For two indicators (QI-9, Contaminated non-microbiological and Contaminated microbiological), laboratory section totals (non-Microbiology and Microbiology, respectively) served as proxies. For the remaining four indicators (clotted sample, hemolysis, incorrect fill volume, and container damaged), the institutional LIS export could not isolate the narrower sub-populations specified by the IFCC (e.g., exclusively anticoagulated or transported tubes); the total received test-order count (905,018) was therefore applied as an over-inclusive proxy denominator (Supplementary File S3).

### 2.7. Stratification and Subgroup Analysis

To identify priority areas for quality improvement, specimen quality failure rates were stratified across four contextual parameters. Rates were calculated per laboratory section using total test orders received in that section as the denominator, and per sample matrix (serum, whole blood, plasma, urine, etc.), restricted to types with sufficient volume (*n* ≥ 30) for meaningful interpretation. Test order records were also stratified by LIS priority designation: “Routine” (standard processing queue) versus “STAT” (urgent queue flagged at order entry) to determine whether urgency influenced collection quality. For work shift, the logged timestamp of rejected test orders was used to classify entries into one of three shifts: Morning (07:30–15:29), Evening (15:30–23:29), and Night (23:30–07:29). As the timestamp data were available only for rejected samples and not for completed samples, shift-specific baseline denominator rates could not be calculated. Therefore, the shift analyses are reported as the relative distribution of rejection events and their cause profiles within each shift. Pareto analysis (80/20 rule) was applied, both globally and within individual laboratory sections, to separate the Vital Few dominant causes from the trivial many.

### 2.8. Statistical Analysis

Descriptive statistics were computed for frequency counts, proportional distributions, cross-tabulations, Sigma metrics, and QI classifications. For key rejection rates and QI proportions, 95% confidence intervals were calculated using the Wilson score method without continuity correction, which provides robust coverage for large denominators and low event rates. Statistical analysis reports were generated with AI assistance and cross-validated in IBM SPSS Statistics version 32.0 (IBM Corp., Armonk, NY, USA).

Given the large number of baseline test orders (*N* = 905,018), formal inferential significance testing (e.g., chi-square) was intentionally omitted. At this scale, even clinically negligible variations in proportion register as statistically significant; differences were instead interpreted using operational effect sizes (absolute and relative differences in rejection rates), Sigma performance, and IFCC benchmarking (Supplementary File S4).

### 2.9. Ethical Considerations

The study protocol was approved by the Research Ethics Committee of Al Rass General Hospital (Approval No. H-04-Q-001, dated 2 July 2025). Given the retrospective, observational nature of this investigation and the exclusive use of de-identified registry data, the ethics committee waived the requirement for individual written informed consent. All data processing and administrative management workflows were executed in strict compliance with institutional ethical frameworks and patient data confidentiality guidelines. During the preparation of this manuscript/study, the authors used Claude Sonnet 5 (Anthropic, San Francisco, CA, USA) for the purposes of data deduplication, categorical classification of laboratory rejection records, manuscript drafting assistance, and reference formatting.

## 3. Results

### 3.1. Overall Rejection Rate and Specimen Quality Failure Rate by Laboratory Section

A total of 905,018 test order records were included across all laboratory sections (Table 1). Of these, 869,542 (96.08%) were completed and 35,476 were rejected, representing an overall rejection rate of 3.92% (95% CI 3.88–3.96%; Sigma 3.26). When rejection events were partitioned using the binary criterion, 19,981 (56.3%) were identified as specimen quality failures, yielding a true quality failure rate of 2.21% (95% CI 2.18–2.24%; Sigma 3.51). The remaining 15,495 rejections (43.7%; 1.71%, 95% CI 1.69–1.74%) were excluded as operational and administrative non-performance events.

Chemistry section accounted for the largest proportion of the total workload (34.36%), followed by Hematology (28.50%) and Hormones & Virology (16.59%). The highest specimen quality failure rate was observed in Hemostasis (3.79%), followed by Parasitology (2.96%). The lowest specimen quality failure rates were seen in POCT (0.20%) and Blood Bank (0.39%).

### 3.2. Pre-Analytical Quality Indicators

The 19,981 pre-analytical specimen quality failures were classified into 15 QI categories (Table 2). The leading cause was clotted samples (QI-2; *n* = 5232; 26.2%), followed by hemolysis (QI-1; *n* = 4599; 23.0%) and wrong sample type (QI-14; *n* = 4042; 20.2%).

The two frameworks, reflecting their distinct evaluative purposes—absolute process capability versus population-relative ranking—produced differing performance classifications for the same underlying data. Against verified IFCC WG-LEPS benchmark percentiles [13], 6 of the 13 benchmarkable QI categories fell Below Minimum despite each achieving a ‘Good’ Westgard Sigma score (≥4.0): clotted sample (0.578% of all samples vs. a threshold of 0.517%), wrong sample type (0.447% vs. 0.022%), insufficient volume (0.157% vs. 0.140%), wrong container (0.059% vs. 0.030%), container damaged (0.050% vs. 0.001%), and non-microbiological contamination (0.037% vs. 0.030%). Hemolysis (0.508%) was within the Desirable band (0.060–0.670%).

### 3.3. Specimen Quality Failure Rate by Sample Type

Serum was the most frequently received sample type, followed by whole blood and plasma (Table 3). Among sample types with a sample size sufficient for meaningful interpretation (*n* ≥ 30), the highest failure rates were observed for sputum (14.81%), iced plasma (13.21%), and body fluids (8.24%). Conversely, plasma bag, segment, and blood-for-culture specimens exhibited the lowest specimen quality failure rates.

### 3.4. Rejection Rate by Clinical Priority (Routine vs. STAT)

Of the total test orders analyzed, 75.87% were submitted as Routine and 24.13% as STAT (Table 4). STAT samples demonstrated a lower pre-analytical specimen quality failure rate than Routine samples (1.25% [95% CI 1.20–1.29%] vs. 2.51% [95% CI 2.48–2.55%]; rate ratio 0.50), indicating that STAT samples failed at roughly half the rate of Routine samples, with correspondingly higher Sigma process capability (3.74 vs. 3.46).

Profiling the specific failure modes revealed distinct transactional patterns between the two priority workflows. Among STAT samples, clotted specimens (QI-2) were the dominant failure mode, accounting for 36.9% of pre-analytical rejections compared to 24.5% in Routine samples. While hemolysis (QI-1) ranked as the second leading cause of rejection in Routine collections (24.1%), it dropped to the third position in STAT collections (16.1%), trailing behind wrong sample type (QI-14; 21.2%).

### 3.5. Rejection Profile by Work Shift and Clinical Priority

The morning shift accounted for the majority of total recorded rejection events (80.98%), followed by the evening shift (11.4%) and the night shift (7.7%). However, when evaluating the underlying drivers under the binary macro-framework, the morning shift demonstrated the lowest relative proportion of specimen-specific issues: only 53.4% of morning rejections were classified as true Pre-Analytical Specimen Quality Failures, compared with 70.2% in the evening shift and 66.3% in the night shift (Table 5). The remaining morning rejections (46.6%) represented Operational and Administrative non-performance events, such as test unavailability, reagent shortages, or IT disruptions, which coincided with the high-volume operational demands characteristic of peak morning hours.

### 3.6. Pareto Analysis and Departmental Rejection Profiles

**Figure 1:**
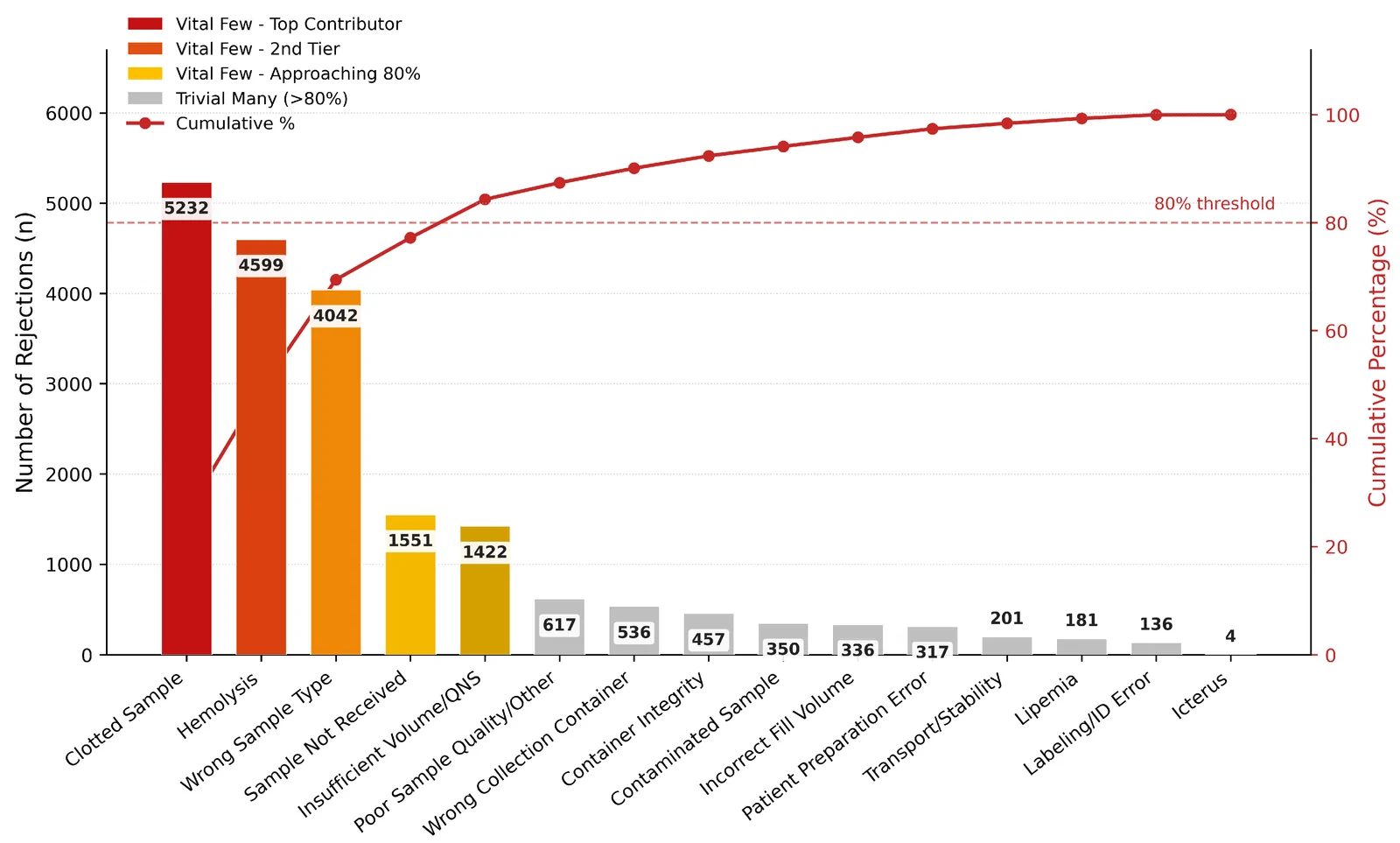
Pareto analysis of pre-analytical quality failure causes (overall; *n* = 19,981). Dashed red lines mark the 80% cumulative threshold separating the Vital Few from the trivial many.

Pareto analysis identified five major QIs as the Vital Few, accounting for 84.3% of all pre-analytical quality failures: clotted samples, hemolysis, wrong sample type, sample not received, and insufficient volume/QNS (Figure 1).

**Figure 2:**
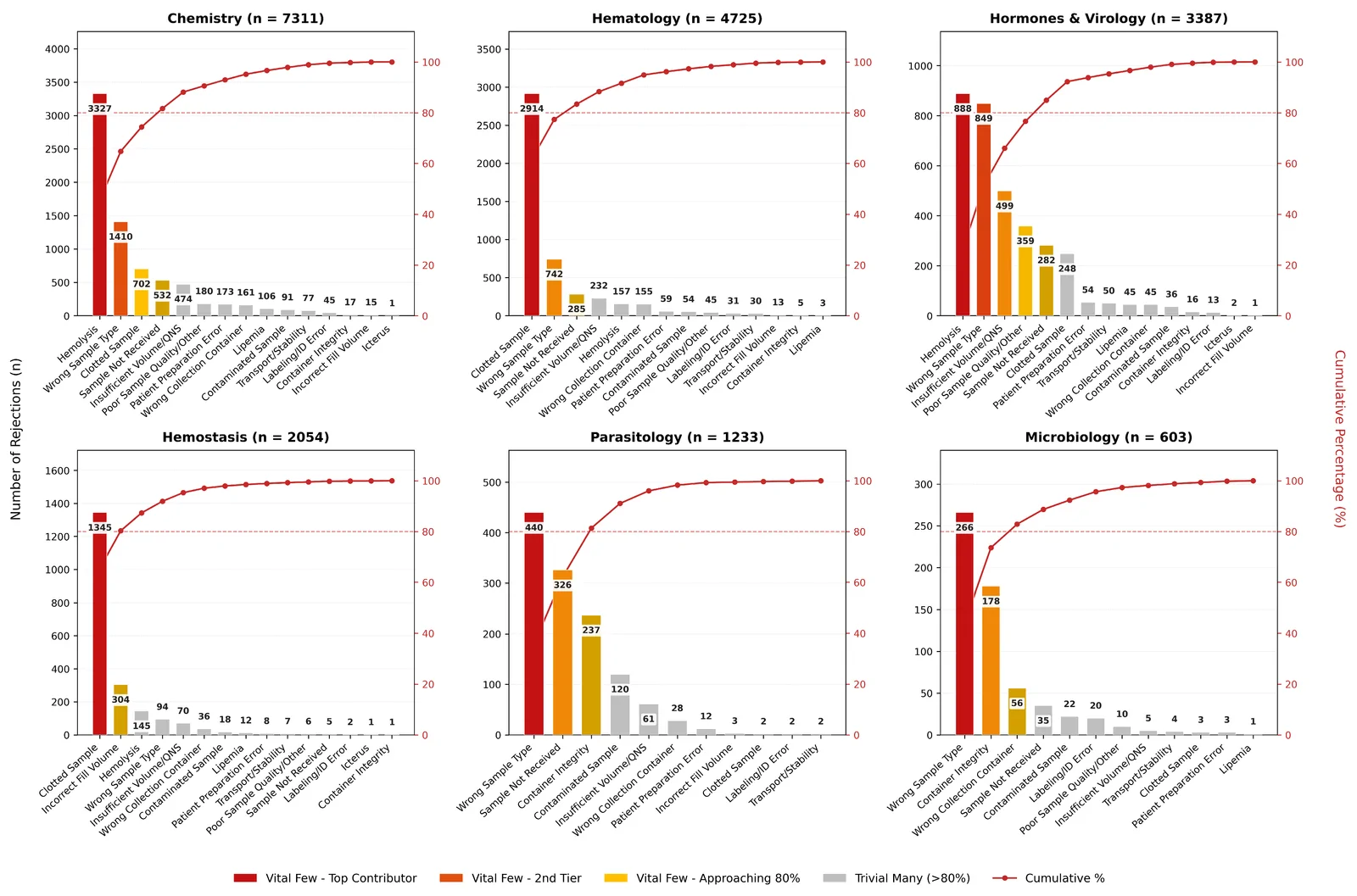
Pareto analysis by laboratory section. Panels show the top pre-analytical quality failure rejection causes for each section, ranked by frequency. Dashed red lines mark the 80% cumulative threshold.

Section-level Pareto analysis revealed markedly different Vital Few profiles across departments (Figure 2). In Chemistry, hemolysis (QI-1) alone accounted for 45.5% of pre-analytical rejections, making it the single dominant cause of specimen failure. Conversely, clotted samples (QI-2; 61.7%) dominated Hematology. For the Hemostasis section, a distinct two-cause profile emerged: clotted specimens (QI-2; 65.5%) and incorrect fill volume (QI-6; 14.8%) together accounted for 80.3% of rejections. Microbiology and Parasitology rejections diverged significantly from these patterns, driven primarily by wrong sample type (QI-14) and container integrity failures (QI-11) rather than phlebotomy-related handling errors.

Table 6 describes the Vital Few quality failure causes across six major laboratory sections. In Chemistry and Hematology, a narrow combination of pre-analytical errors, primarily clotted samples, hemolysis, wrong samples collected, and unreceived specimens, accounts for 81.7% and 83.4% of their total rejections, respectively. Importantly, Hemostasis demonstrates that just two specific categories (clotted samples and incorrect fill volumes) drive 80.3% of its entire pre-analytical quality failure burden. Hormones & Virology presents a more distributed pattern requiring five distinct categories. The lower-volume areas of Parasitology and Microbiology are uniquely vulnerable to collection container issues and sample misidentification.

## 4. Discussion

### 4.1. Overall Rejection Rate in Context

This two-year audit identified an overall Pre-Analytical Specimen Quality Failure rate of 2.21% (Sigma 3.51), representing 19,981 (56.3%) of the 35,476 total recorded rejections; the remaining 15,495 (43.7%) were classified as Operational and Administrative Non-Performance Events. The aggregated rejection rate would have been reported as 3.92%, nearly double the partitioned figure. This sensitivity is the study’s central methodological finding: the headline rejection rate depends entirely on how non-pre-analytical rejection causes are handled. The 2.21% figure is not directly comparable to most published benchmarks, including the pooled global estimate of 1.99% [7], the rate reported in a Saudi tertiary-care audit (2.25%) [11], and single-site audits [9,16] because these studies report all-cause rejection rates without distinguishing specimen-quality failures from operational and administrative causes, precisely the conflation this study set out to resolve.

This incomparability echoes a broader concern that the overall rejection rate, despite its status as a near-universal quality indicator, is calculated and defined inconsistently across studies [17], and reinforces the recommendation that future audits report not only an aggregate rate but also an explicit partitioning of cause categories, consistent with IFCC WG-LEPS guidance. The partitioned pre-analytical specimen quality failure rate of 2.21% should therefore be read not as a favorable comparison to prior benchmarks, but as a demonstration of the method itself: a laboratory’s true specimen-handling performance can only be assessed once operational and administrative causes are removed.

### 4.2. Hemolysis and Clotted Specimens as the Dominant Causes

Clotted samples (QI-2; 26.2%) and hemolysis (QI-1; 23.0%) were the two leading individual causes identified in this study, together accounting for nearly half of all specimen quality failures. This pattern mirrors findings from Saudi Arabia [11], Ethiopia [10], and the global meta-analytic estimate [7]. It is consistent with the well-established mechanistic link between hemolysis and inappropriate phlebotomy technique, including the use of an undersized needle with excessive vacuum, vigorous aspiration through a syringe, or prolonged tourniquet application [18]. Clotted specimens, similarly, are attributable in the overwhelming majority of cases to inadequate or delayed mixing of the sample with anticoagulant immediately following venipuncture [19].

### 4.3. Divergent Performance Signals from Internal Sigma Metrics and External IFCC Benchmarks

This study extends the existing literature by applying Westgard Six Sigma metrics and IFCC WG-LEPS benchmarking simultaneously, highlighting how their differing evaluative concepts provide distinct operational perspectives. The Westgard Six Sigma model is an absolute metric anchored to a universal benchmark, measuring intrinsic process capability. In contrast, the IFCC model adopts a population-relative framework based on the distribution of performance across participating peer laboratories, where the 25th, 50th, and 75th percentiles define optimal, desirable, and minimum performance levels.

Consequently, the differing classifications against the two tools reflect their distinct purposes. Against verified IFCC peer percentiles, six of the thirteen benchmarkable QI categories fell into the ‘Below Minimum’ band (placing them in the bottom quartile of participating international laboratories) despite each achieving an internally ‘Good’ Westgard Sigma score (≥4.0): clotted sample (0.578% vs. a 0.517% threshold), wrong sample type (0.447% vs. 0.022%), insufficient volume (0.157% vs. 0.140%), wrong container (0.059% vs. 0.030%), container damaged (0.050% vs. 0.001%), and non-microbiological contamination (0.037% vs. 0.030%). Hemolysis (0.508%), by contrast, falls within the Desirable band (0.060–0.670%).

Evaluating either framework in isolation provides an incomplete picture: relying solely on absolute Sigma scores may obscure the fact that an error rate ranks unfavorably relative to international peers, whereas relying solely on peer percentiles omits the perspective of absolute defect minimization [20]. Applying both frameworks in tandem provides a more balanced assessment of laboratory quality.

### 4.4. Departmental Heterogeneity in Specimen Quality Failure Profiles

The present findings illustrate distinct specimen quality failure profiles across laboratory sections. In Chemistry, hemolysis (QI-1) was the single dominant cause (45.5%), consistent with the section’s reliance on serum and plasma assays that are particularly susceptible to interference from free hemoglobin [18]. In Hematology, by contrast, clotted specimens (QI-2) accounted for 61.7% of rejections, reflecting the section’s dependence on EDTA-anticoagulated whole blood for complete blood counts.

The Hemostasis section displayed a uniquely narrow, two-cause profile: clotted specimens (QI-2; 65.5%) and incorrect fill volume (QI-6; 14.8%) together accounted for 80.3% of section-level rejections. This finding is mechanistically well explained by the strict requirement that citrate-anticoagulated coagulation specimens be filled to within approximately 10% of the 9-to-1 blood-to-citrate ratio; both under- and over-filling distort prothrombin time and activated partial thromboplastin time results [21].

Microbiology and Parasitology displayed distinct profiles from the blood-based sections, dominated by container integrity failures (QI-11) and wrong sample type (QI-14) rather than venipuncture-related causes. This is consistent with the heterogeneous and often patient-collected nature of microbiological and parasitological specimens (swabs, stool, and body fluids), where inappropriate containers, transport damage, and delayed transit, rather than phlebotomy technique, represent the principal points of failure [22].

### 4.5. The STAT Paradox and Shift Dynamics

In our data, STAT samples demonstrated a lower specimen quality failure rate than Routine collections (1.25% vs. 2.51%; 3.74σ vs. 3.46σ). This observation contrasts with several published reports where urgent, high-acuity clinical environments were associated with higher error rates [23,24]. Various non-exclusive operational factors may help explain this pattern. First, prioritized STAT workflows typically feature accelerated transit and immediate laboratory accessioning, consistent with reduced transit-related delays, minimized tube standing times, and attenuated in vitro sample degradation. Second, variation in staffing composition across clinical units or shifts may contribute; for example, if urgent collections are disproportionately performed by experienced acute-care personnel, this could influence baseline sample integrity. However, because our LIS dataset lacked tracking of individual collector identities, ward-level staffing models, or time-to-centrifugation intervals, these operational mechanisms remain plausible working hypotheses rather than established causal factors, warranting dedicated prospective investigation.

Clotting (QI-2) accounted for 36.9% of STAT specimen quality failures (compared to 24.5% in Routine). This narrower failure signature makes the STAT error profile highly actionable via targeted mixing compliance protocols in acute care units, whereas Routine workflows require broader, system-wide workforce retraining.

Similarly, the temporal distribution of specimen quality failure rates requires cautious interpretation. Although the morning shift accounted for the majority of total logged rejections (80.98%), only 53.4% of these events represented true specimen quality failures. This shift-level variation must be viewed as a descriptive association rather than evidence of shift-induced collection errors. In our setting, work shift and specimen processing volume are inherently confounded: morning hours coincide with major inpatient phlebotomy rounds, outpatient clinic surges, batch order entries, and routine maintenance schedules. Because baseline denominator volume for completed specimens was not logged by shift, we cannot decouple a genuine shift effect from routine volume loading, nor can we assign causality to specific operational bottlenecks based on retrospective registry logs alone.

### 4.6. Implications for Quality Improvement

The Pareto analysis identified five primary categories (QI-1, QI-2, QI-5, QI-10, and QI-14) that drive 84.3% of all specimen quality failures, offering a data-backed blueprint for resource allocation. However, the literature shows that addressing these Vital Few requires structural rather than purely didactic interventions. While barcode-based patient identification and automated bedside labeling consistently minimize clerical misidentification [25], simple classroom-based phlebotomy retraining often yields negligible long-term improvements in clotting or hemolysis rates [26].

Durable process optimization requires structural, closed-loop changes. Effective strategies include deploying dedicated phlebotomy teams to high-risk clinical zones, updating standard collections with advanced blood-draw devices, and establishing unit-level dashboards that report rejection metrics directly back to department managers [5,23].

### 4.7. Study Limitations

Several limitations should be acknowledged when interpreting these findings. First, this is a retrospective, single-center study; the results may not be generalized across institutions. Second, the primary unit of analysis is the test/exam-order record rather than unique physical specimens, as the LIS architecture did not record container-level identifiers for completed tests to permit specimen-level deduplication. Third, because the dataset lacked granular tracking regarding individual collector identity, the plausible collector identity hypothesis for the STAT paradox cannot be substantiated. Fourth, this study is limited by using the 2017–2018 IFCC WG-LEPS dataset for comparison, which may not capture recent longitudinal shifts. In this context, routine participation in the IFCC Quality Indicators project provides enrolled laboratories with ongoing, dynamic peer comparison with real-time contextualization that our retrospective design cannot offer. To address this temporal gap, we conducted a sensitivity analysis against the updated 2021 cycle. Our primary conclusions proved highly robust: 11 of the 12 benchmarkable quality indicators were classified identically across both cycles. The sole shifting indicator was Excessive Transport Time (Pre-ExcTim; observed rate: 0.017%), which was classified as Desirable under the 2018 benchmark (threshold: 0.035%) but moved to Below Minimum under the tighter 2021 threshold (0.010%), adjusting our headline summary from 6 of 13 to 7 of 13 categories falling Below Minimum. Nonetheless, we retained the 2017–2018 dataset as our primary baseline because the 2021 cycle was gathered during the COVID-19 pandemic. The source authors explicitly acknowledged introducing atypical operational strains, incomplete submissions, and shifting laboratory cohorts, unlike the stable, non-pandemic conditions characterizing our study period. Finally, an important methodological consideration involves the denominators used for external IFCC benchmarking. While nine indicators utilized identical or proxy-adjusted denominators consistent with IFCC definitions, four indicators (clotted sample, hemolysis, incorrect fill volume, and container damaged) relied on total received samples because our LIS could not isolate specific sub-cohorts (such as anticoagulated or transported specimens). This introduces a known, unidirectional conservative bias that understates the true failure rate. For clotted sample (QI-2) and container damaged (QI-11), our conclusions are robust because both indicators were Below Minimum despite this conservative calculation. In contrast, for hemolysis (QI-1, Desirable) and incorrect fill volume (QI-6, Optimal), this constraint introduces residual interpretive uncertainty, as evaluating these failures against their true, narrower denominators could plausibly shift their classifications into less favorable tiers.

## 5. Conclusions

By excluding 15,495 Operational and Administrative Events from the data, the study isolated 19,981 Specimen Quality Failures with a rate of 2.21% (σ = 3.51). Pareto analysis identified clotted samples, hemolysis, wrong sample type, samples not received, and insufficient volume as dominant causes of specimen quality failures. Furthermore, failure modes differed substantially across departments, suggesting that uniform, laboratory-wide interventions may fail to address the specific operational realities of individual sections.

Additionally, evaluating internal Westgard Sigma metrics alongside international IFCC WG-LEPS benchmarks demonstrated the value of combining absolute process capability with peer-referenced standards. While six of thirteen benchmarked QIs, including clotted specimens, wrong sample type, insufficient volume, wrong container, container damaged, and non-microbiological contamination, achieved satisfactory absolute Sigma scores above 4.0, peer benchmarking contextualized these findings by showing that they simultaneously fell into the bottom quartile (Below Minimum) of international peer performance. Furthermore, the audit observed an unexpected ‘STAT paradox,’ wherein urgent samples exhibited a lower specimen quality failure rate than routine collections (1.25% vs. 2.51%), highlighting an operational pattern that warrants prospective mechanistic investigation across differing clinical staffing models.

These findings support a shift in how laboratory quality is monitored and managed. First, future laboratory rejection audits should separate specimen quality failures from operational and administrative events, since conflating the two distorts cross-institutional comparisons and obscures localized clinical accountability and targeted interventions. Second, internal Sigma metrics should be cross-referenced against external quality specifications to help maintain international clinical safety standards. Last, institutional quality improvement frameworks would benefit from moving away from generic, blanket policies toward more targeted interventions designed around the specific intersections of laboratory sections, specimen types, clinical priority tiers, and work shifts.

## Figures and Tables

**Table 1:** Pre-analytical Specimen Quality Failure Rate by Laboratory Section.

Section	Total Samples	% of Total	Failures, *n*	Failures, %	Sigma
Chemistry	310,963	34.36%	7311	2.35%	3.49
Hematology	257,896	28.50%	4725	1.83%	3.59
Hormones & Virology	150,142	16.59%	3387	2.26%	3.50
Hemostasis	54,160	5.98%	2054	3.79%	3.28
Parasitology	41,720	4.61%	1233	2.96%	3.39
POCT	31,422	3.47%	63	0.20%	4.38
Microbiology	27,287	3.02%	603	2.21%	3.51
Special Proteins/Tumor Markers	11,697	1.29%	297	2.54%	3.45
Serology	8667	0.96%	180	2.08%	3.54
Blood Bank	6382	0.71%	25	0.39%	4.16
Histopathology	4477	0.49%	93	2.08%	3.54
Drugs/Toxicology	205	0.02%	10	4.88%	3.16
Total	905,018	100%	19,981	2.21% *	3.51 *

Sigma calculated using the Westgard method (DPM conversion with 1.5 process shift). Westgard tiers: World Class ≥ 6.0; Excellent 5.0–5.9; Good 4.0–4.9; Acceptable 3.0–3.9; Needs Improvement < 3.0. * Reported as the pooled specimen quality failure rate (19,981/905,018).

**Table 2:** Specimen Quality Failures by Quality Indicators (QIs): Frequency, Sigma Performance, and IFCC Benchmarking.

Code (IFCC)	Category	*n*	% of Specimen Quality Failure	% of All Test Orders	95% CI (%)	Sigma	Westgard	IFCC Level
QI-2 (Pre-Clot)	Clotted Sample	5232	26.2	0.578 †	0.563–0.594	4.03	Good	Below Minimum
QI-1 (Pre-HemR)	Hemolysis (rejected)	4599	23.0	0.508 †	0.494–0.523	4.07	Good	Desirable
QI-14 (Pre-WroTy)	Wrong Sample Type	4042	20.2	0.447	0.433–0.461	4.11	Good	Below Minimum
QI-10 (Pre-NotRec)	Sample Not Received	1551	7.8	0.171	0.163–0.180	4.43	Good	Desirable
QI-5 (Pre-InsV)	Insufficient Volume	1422	7.1	0.157	0.149–0.165	4.45	Good	Below Minimum
QI-15	Poor Sample Quality/Other	617	3.1	0.068	0.063–0.074	4.70	Good	N/A
QI-7 (Pre-WroCo)	Wrong Container	536	2.7	0.059	0.054–0.065	4.74	Good	Below Minimum
QI-11 (Pre-DamS)	Container Damaged	457	2.3	0.050 †	0.046–0.055	4.79	Good	Below Minimum
QI-6 (Pre-SaAnt)	Incorrect Fill Volume	336	1.7	0.037 †	0.033–0.041	4.87	Good	Optimal
QI-9 (Pre-Cont)	Contaminated (non-micro)	328	1.6	0.037 ‡	0.034–0.042	4.88	Good	Below Minimum
QI-13	Patient Preparation Error	317	1.6	0.035	0.031–0.039	4.89	Good	N/A
QI-3	Lipemia	181	0.9	0.020	0.017–0.023	5.04	Excellent	N/A
QI-12 (Pre-ExcTim)	Excessive Transport Time	156	0.8	0.017	0.015–0.020	5.08	Excellent	Desirable
QI-8 (Pre-MisS)	Labeling/ID Error	136	0.7	0.015	0.013–0.018	5.11	Excellent	Desirable
QI-12 (Pre-InTem)	Wrong Transport Temp	44	0.2	0.005	0.004–0.007	5.40	Excellent	Desirable

† Calculated using total received test records as a proxy denominator. ‡ Calculated against the non-Microbiology section total as an IFCC-specified proxy denominator. 95% CIs were calculated using the Wilson score method without continuity correction. Sigma scores were calculated using the Westgard method (DPM conversion with 1.5 process shift). N/A = no published IFCC specification available. Categories with *n* < 30 (Contaminated/microbiological, *n* = 22; Icterus, *n* = 4; and an unclassified Transport/Stability case, *n* = 1) are not included.

**Table 3:** Pre-Analytical Specimen Quality Failure Rate by Sample Type.

Sample Type	Total Samples	% of All Received	Rejections, *n* (%)	Sigma	Westgard
Serum	434,764	48.04%	9634 (2.22%)	3.51	Acceptable
Whole Blood	331,608	36.64%	6008 (1.81%)	3.59	Acceptable
Plasma	54,174	5.99%	2056 (3.80%)	3.27	Acceptable
Urine	37,630	4.16%	785 (2.09%)	3.54	Acceptable
Stool	10,942	1.21%	689 (6.30%)	3.03	Acceptable
Swab	10,354	1.14%	109 (1.05%)	3.81	Acceptable
Urine Clean Catch	8626	0.95%	278 (3.22%)	3.35	Acceptable
Blood for Culture	4976	0.55%	18 (0.36%)	4.19	Good
Tissue for Pathology	4423	0.49%	91 (2.06%)	3.54	Acceptable
Segment	1709	0.19%	7 (0.41%)	4.14	Good
Plasma Bag	1623	0.18%	0 (0.00%)	6.00	World Class
Iced Plasma	780	0.09%	103 (13.21%)	2.62	Needs Improvement
24-Hr. Urine	768	0.08%	33 (4.30%)	3.22	Acceptable
Sputum	601	0.07%	89 (14.81%)	2.54	Needs Improvement
Seminal Fluid	589	0.07%	19 (3.23%)	3.35	Acceptable
Tracheal Aspirate	511	0.06%	4 (0.78%)	3.92	Acceptable
Body Fluids	376	0.04%	31 (8.24%)	2.89	Needs Improvement
CSF	325	0.04%	10 (3.08%)	3.37	Acceptable
Other	136	0.02%	7 (5.15%)	3.13	Acceptable

Sigma calculated using the Westgard Six Sigma method (DPM conversion with 1.5 process shift). Westgard tiers: World Class ≥ 6.0; Excellent 5.0–5.9; Good 4.0–4.9; Acceptable 3.0–3.9; Needs Improvement < 3.0. CSF = cerebrospinal fluid.

**Table 4:** Pre-Analytical Rejection Profile: Routine vs. STAT.

Priority	Total Samples	All Rejections, *n* (%)	Specimen Quality Failures, *n* (%)	Sigma Score	Top 3 Causes (% of Specimen Quality Failures)
Routine	686,567	30,897 (4.50%)	17,260 (2.51%)	3.46	1. Clotted Sample (24.5%) 2. Hemolysis (24.1%) 3. Wrong Sample Type (20.1%)
STAT	218,451	4579 (2.10%)	2721 (1.25%)	3.74	1. Clotted Sample (36.9%) 2. Wrong Sample Type (21.2%) 3. Hemolysis (16.1%)
Total	905,018	35,476 (3.92%)	19,981 (2.21%)	3.51	—

STAT = Short Turnaround Time. “All Rejections” encompasses total recorded events, including both specimen quality failures and operational events causes. Sigma Score and the Top 3 causes are based on specimen quality failures.

**Table 5:** Total and Quality Failure Rejection Profile by Work Shift and Clinical Priority.

Shift	Total Rejections	Specimen Quality Failures, *n* (% of Shift)	Operational and Administrative Events, *n* (% of Shift)	STAT, *n* (% of Shift)	Top 3 Pre-Analytical Causes (% of Specimen Quality Failures)
Morning	28,720 (80.98%)	15,344 (53.4%)	13,376 (46.6%)	2279 (7.9%)	1. Clotted Sample (25.1%) 2. Hemolysis (24.1%) 3. Wrong Sample Type (23.8%)
Evening	4032 (11.4%)	2831 (70.2%)	1201 (29.8%)	1319 (32.7%)	1. Clotted Sample (33.3%) 2. Hemolysis (20.2%) 3. Sample Not Received (12.9%)
Night	2724 (7.7%)	1806 (66.3%)	918 (33.7%)	981 (36.0%)	1. Clotted Sample (24.1%) 2. Sample Not Received (19.4%) 3. Hemolysis (18.3%)
Total	35,476 (100%)	19,981 (56.3%)	15,495 (43.7%)	4579 (12.9%)	—

Shift-specific rejection rates per total samples cannot be calculated as timestamps are available only for rejected samples. STAT % = proportion of each shift’s rejections that were STAT priority.

**Table 6:** Summary of Vital Few Categories by Laboratory Section.

Section	Quality Failures	Vital Few Categories	Cumulative %
Chemistry	7311	Hemolysis; Wrong Sample Type; Clotted Sample; Sample Not Received	81.7%
Hematology	4725	Clotted Sample; Wrong Sample Type; Sample Not Received	83.4%
Hemostasis	2054	Clotted Sample; Incorrect Fill Volume	80.3%
Hormones & Virology	3387	Hemolysis; Wrong Sample Type; QNS; Poor Sample Quality; Sample Not Received	84.9%
Microbiology	603	Wrong Sample Type; Container Integrity; Wrong Collection Container	82.9%
Parasitology	1233	Wrong Sample Type; Sample Not Received; Container Integrity	81.3%

## Data Availability

The data that support the findings of this study were retrieved from Al Rass General Hospital, Qassim Health Cluster, KSA. Restrictions apply to the availability of these data, which were used under institutional authorization for the current study and so are not publicly available. De-identified data are, however, available from the corresponding author upon request.

## Supplementary Materials

The following supporting information can be downloaded at: https://www.mdpi.com/article/10.3390/jcm15197663/s1, File S1: Supplementary_File_S1_LIS_data_file_coded_without ID; File S2: Supplementary_File_S2_calculating_Sigma values; File S3: Supplementary_File_S3_IFCC_Verification; File S4: Supplementary_File_S4_Consolidated_Methods_M1.
