# Supplementary material for: Partitioning All-Cause Pre-Analytical Laboratory Rejections: A 25-Month Retrospective Audit of Specimen Quality Failures with Six Sigma, IFCC, and Pareto Analysis
Source: J Clin Med. 2026 Oct 3;15(19):7663. doi: 10.3390/jcm15197663 (PMC13666386; doi:10.3390/jcm15197663)
Supplement: Supplementary file 1 [file jcm-15-07663-s001.zip › Supplementary_File_S3_IFCC_Verification.pdf]

Supplementary File S3

IFCC WG-LEPS Benchmark Assessment — Formulae, Numerators, and Denominators, Against the Source Article

Source: Sciacovelli L, Lippi G, Sumarac Z, et al. (IFCC WG-LEPS). "Pre-analytical quality indicators in laboratory medicine: Performance of laboratories participating in the IFCC working group 'Laboratory Errors and Patient Safety' project." Clinica Chimica Acta 497 (2019) 35–40, Tables 1–2.

IFCC Code	QI (this study)	Numerator (per source article)	Denominator (per source article)	Numerator used (this study)	Denominator used (this study)	Rate	Thresholds High/Low (%)	IFCC Level
†Pre-Clot	Clotted Sample (QI-2)	Samples clotted	Samples with an anticoagulant, checked for clots	Samples clotted (n=5,232)	ALL samples (905,018) — narrower denominator not available in source data	0.578%	0.117 / 0.517	Below Minimum
†Pre-HemR	Hemolysis (QI-1)	Samples rejected due to hemolysis	Samples checked for hemolysis	Hemolysis rejections (n=4,599)	ALL samples (905,018) — narrower denominator not available	0.508%	0.060 / 0.670	Desirable
Pre-WroTy	Wrong Sample Type (QI-14)	Samples of wrong/inappropriate type	Total samples	Wrong-type rejections (n=4,042)	ALL samples (905,018) — matches article	0.447%	0.000 / 0.022	Below Minimum
Pre-NotRec	Sample Not Received (QI-10)	Samples not received	Total samples	Not-received rejections (n=1,551)	ALL samples (905,018) — matches article	0.171%	0.090 / 1.110	Desirable
Pre-InsV	Insufficient Volume (QI-5)	Samples with insufficient volume	Total samples	Insufficient-volume rejections (n=1,422)	ALL samples (905,018) — matches article	0.157%	0.020 / 0.140	Below Minimum
Pre-WroCo	Wrong Container (QI-7)	Samples collected in wrong container	Total samples	Wrong-container rejections (n=536)	ALL samples (905,018) — matches article	0.059%	0.000 / 0.030	Below Minimum
†Pre-SaAnt	Incorrect Fill Volume (QI-6)	Samples with inappropriate sample–anticoagulant ratio	Samples with anticoagulant	Fill-volume rejections (n=336)	ALL samples (905,018) — narrower denominator not available	0.037%	0.095 / 0.855	Optimal
‡Pre-Cont	Contaminated, non-micro (QI-9)	Contaminated samples rejected	Non-microbiological samples	Non-micro contamination (n=328)	Non-Microbiology-section total (877,731)	0.037%	0.003 / 0.030	Below Minimum
‡Pre-MicCon	Contaminated, micro (QI-9)	Microbiological contaminated samples rejected	Microbiological samples	Micro contamination (n=22)	Microbiology-section total (27,287)	0.081%	0.620 / 1.530	Optimal
†Pre-DamS	Container Damaged (QI-11)	Samples damaged during transportation	Transported samples	Damaged-container rejections (n=457)	ALL samples (905,018) — narrower denominator not available	0.050%	0.000 / 0.001	Below Minimum

Pre-InTem	Wrong Transport Temp (QI-12)	Samples transported at inappropriate temperature	Total samples	Wrong-temp rejections (n=44)	ALL samples (905,018) — matches article	0.005%	0.000 / 1.245	Desirable
Pre-ExcTim	Excessive Transport Time (QI-12)	Samples with excessive transportation time	Total samples	Excess-time rejections (n=156)	ALL samples (905,018) — matches article	0.017%	0.000 / 0.035	Desirable
Pre-MisS	Labeling/ID Error (QI-8)	Misidentified samples	Total samples	Mislabeling rejections (n=136)	ALL samples (905,018) — matches article	0.015%	0.000 / 0.041	Desirable

- All 13 High/Low threshold pairs were re-checked character-by-character against the source article's Table 2 (2018 laboratory performance data) and matched exactly; no threshold values required correction.
- †The source article specifies a denominator narrower than “all samples received” (e.g., only samples with anticoagulant, only samples checked for hemolysis, only transported samples). The raw LIS export used for this study does not carry a flag identifying which samples fall into these narrower populations, so the total sample count (905,018) was used as the denominator for these four indicators instead — an approximation, not an oversight.
- ‡Two of the six narrower-denominator indicators COULD be computed with an available proxy — Microbiology-section total sample count as a stand-in for “microbiological samples,” and non-Microbiology-section total as a stand-in for “non-microbiological samples.”
- Directional implication for the four indicators that could not be recomputed (Pre-Clot, Pre-HemR, Pre-SaAnt, Pre-DamS): using the total sample count instead of the article's narrower intended denominator produces a SMALLER computed rate than the true indicator value, since the numerator is being divided by a larger-than-intended population. Recomputing with the correct (narrower) denominator would only increase these rates — moving them toward, not away from, “Below Minimum” classification.
- Pre-Clot and Pre-DamS are already classified Below Minimum under the current (conservative) denominator; a corrected, narrower denominator cannot change this classification since it can only push the rate higher, not lower.
- Pre-HemR (currently Desirable) and Pre-SaAnt (currently Optimal) are the two indicators where a corrected denominator could plausibly move the classification into a worse band — these are the two to flag for readers as having the most classification uncertainty under the current approximation.
