# Supplementary material for: Partitioning All-Cause Pre-Analytical Laboratory Rejections: A 25-Month Retrospective Audit of Specimen Quality Failures with Six Sigma, IFCC, and Pareto Analysis
Source: J Clin Med. 2026 Oct 3;15(19):7663. doi: 10.3390/jcm15197663 (PMC13666386; doi:10.3390/jcm15197663)
Supplement: Supplementary file 1 [file jcm-15-07663-s001.zip › Supplementary_File_S4_Consolidated_Methods_M1.pdf]

Supplementary File S4 – Consolidated Data Analysis Methods

Complete record of every data-processing and analytical step applied to the LIS rejection dataset, across all stages of this project

1. Overview

This document consolidates every methodological step performed on the LIS dataset in support of Pre-Analytical Specimen Quality Failures. It combines the individual methods notes produced at each stage (classification, IFCC benchmarking, sample-type consolidation) into a single reference. Final dataset: 905,018 records (869,542 completed test orders, 35,476 rejected test-order entries).

2. Data Source and Extraction

Data were extracted electronically from the institutional Laboratory Information System (LIS) at Al Rass General Hospital, covering 25 months (July 2023 – August 2025). The raw export contained one row per test/exam order (not per physical specimen), with fields: Case ID, Patient ID, Reject DateTime, Reject Reason, Sample Type, Collection Status, Section, Priority, and Exam.

3. Classification: Group 1 vs. Group 2

All 35,476 rejection entries were classified using a deterministic, AI-assisted mapping dictionary (Claude Sonnet 5), not per-record prompting. The 98 distinct raw Reject Reason strings present in the data were each reviewed once and assigned a category; this mapping was then applied programmatically across all 35,476 rows via a vectorized lookup.

- Binary partitioning criterion: would a perfectly collected, perfectly handled specimen still have been rejected for this reason? ‘No’ → Group 1 (Pre-Analytical Specimen Quality Failure). ‘Yes’ → Group 2 (Operational and Administrative Non-Performance).
- Match basis tracked per raw string: ‘listed’ = verbatim match to a representative example in the supplied classification framework (35,021 rows, 98.7%); ‘judgment’ = mapped using the framework's stated inclusion logic where no example matched exactly (455 rows, 1.3%, spanning 38 raw-text variants).
- Specimen Quality Failure entries were further mapped to the 15 standardized IFCC WG-LEPS quality indicator (QI) categories.

4. IFCC Benchmark Levels

QI category rates were benchmarked against verified, published IFCC WG-LEPS quality specifications: Sciacovelli L, Lippi G, Sumarac Z, et al. “Pre-analytical quality indicators in laboratory medicine: Performance of laboratories participating in the IFCC working group ‘Laboratory Errors and Patient Safety’ project.” Clinica Chimica Acta 497 (2019) 35-40, Table 2. High/Medium/Low thresholds verified against this source for all 13 benchmarkable categories; matched exactly.

- QI-9 (Contaminated) was split by Section into Pre-Cont (non-microbiological) vs. Pre-MicCon (microbiological, Section = Microbiology).
- QI-12 (Transport/Stability) was split by raw-text keyword into Pre-InTem (temperature-related) vs. Pre-ExcTim (time/staleness-related); 1 row (‘light exposure’) fit neither and is unclassified against this benchmark.
- 3 categories with $n<30$ (Contaminated/microbiological $n=22$, Icterus $n=4$, unclassified Transport/Stability $n=1$) are excluded from the displayed Table 2, consistent with the $n\geq30$ convention used in Table 3, with a footnote; these 27 rows remain in the Group 1 total.
- Result: 6 of 13 benchmarkable categories fall Below Minimum despite each achieving a ‘Good’ Westgard Sigma score (≥4.0): Clotted Sample, Wrong Sample Type, Insufficient Volume, Wrong Container, Container Damaged, Contaminated (non-micro). Hemolysis is within the Desirable band, not at a minimum-threshold boundary.

5. Sample Type Consolidation

The raw 'Sample Type' field contained 62 distinct values, including spelling/data-entry variants and section-dependent ambiguities. Consolidated to 19 reportable categories (n≥30) using this precedence order:

5.1 Section-priority rule (applied first)

Any sample logged under the Pathology section → "Pathology", except body-fluid types → "Body Fluids" instead. This absorbed Tissue, Tissue Unspecified, Appendix, Excision, Biopsy, Slide, and Pathology-section 'Other' rows into one category (n=4,423).

5.2 Section-conditional types

- Skin → Swab if Microbiology, else Other (empirically 100% Microbiology, n=51).
- Abscess → Swab if Microbiology, else Other (empirically 100% Microbiology, n=149).

5.3 Fixed spelling/synonym merges

- Serum = Serum + Serum. + Plasma. (The Plasma rows are confirmed glucose/lactate plasma-tube draws from Chemistry — Exam = Glucose 2H PP / Lactate — not a data-entry error; merge confirmed correct by author).
- Plasma = Plasma (kept separate from Serum).
- Whole Blood = Whole Blood + Whole Blood-Malaria + Whole Blood-Pediatric.
- Blood for Culture = Whole Blood-Aerobic + Whole Blood-Anaerobic.
- Urine = Urine + Random Urine.
- Stool = Stool = Fecal + Stool.
- Body Fluids = Body Fluid, Unsp + Synovial Fluid (Joint Fluid) + Pleural Fluid (Thoracentesis Fld) + Fluid + plain Body Fluid + Pathology-section body-fluid rows.
- Swab = Axilla Swab, Nasal, Groin Swab, Nasal Swab, Swab, Ear Swab, Throat Swab, Wound Swab, Nasal-Throat Swab, Umbilical Swab, Wound + Skin (Microbiology) + Abscess (Microbiology).
- Other = Other (non-Pathology portion) + Platelet Poor Plasma + Blood Bag.

Everything else (Urine Clean Catch, Segment, Plasma Bag, Iced Plasma, 24Hr Urine, Sputum, Seminal Fluid, Tracheal Aspirate, CSF, etc.) kept its own category, unchanged.

6. Six Sigma Metrics

Sigma = $\Phi^{-1}(1 - \text{defect rate}) + 1.5$ (standard long-term, 1.5σ-shifted conversion; Φ^{-1} = inverse standard normal CDF). Displayed Sigma capped at 6.0 when the raw calculation exceeds it (standard convention, matching the cited IFCC source methodology). Westgard tiers: World Class ≥6.0; Excellent 5.0–5.9; Good 4.0–4.9; Acceptable 3.0–3.9; Needs Improvement <3.0.

7. Pareto Analysis

Categories ranked by descending frequency; cumulative % computed; "vital few" = the smallest leading set of categories whose cumulative percentage first reaches or exceeds 80% (literal 80/20 rule). Applied overall and per major section (Group 1 n≥100).

8. Validation

- Classification accuracy: manual re-review of a random 10% audit sample, stratified by 15 QI category and 98 distinct raw rejection-reason strings, by two independent authors against the master framework definitions — 100% accuracy.

- Reproducibility: the entire data-cleaning and classification pipeline was re-executed in three independent, isolated sessions; only minor, non-material differences were observed across sessions, attributable predominantly to the deduplication step rather than the classification mapping itself.

9. Statistical Approach

No formal inferential hypothesis testing (e.g., chi-square) was performed, given the very large sample size (N = 905,018), at which even trivial differences in proportion register as statistically significant. All comparisons are descriptive: frequencies, percentages, rate differences, and Sigma metrics. For key rejection rates and QI proportions, 95% confidence intervals were calculated using the Wilson score.
